# The effects of antenatal corticosteroid exposure on the rate of hyperbilirubinemia in term newborns

**DOI:** 10.12669/pjms.35.6.1218

**Published:** 2019

**Authors:** Ilknur Col Madendag, Mefkure Eraslan Sahin

**Affiliations:** 1 Ilknur Col Madendag, MD, Department of Obstetrics and Gynecology, Health Sciences University Kayseri, City Hospital, Kayseri, Turkey; 2 Mefkure Eraslan Sahin, MD, Department of Obstetrics and Gynecology, Health Sciences University Kayseri, City Hospital, Kayseri, Turkey

**Keywords:** Antenatal corticosteroid, Betamethasone, Neonatal hyperbilirubinemia, Term pregnancy

## Abstract

**Objective:**

Neonatal hyperbilirubinemia is a short-lasting benign condition that affects approximately 60% of infants born at term infants. This study aimed to evaluate the effects of antenatal corticosteroid (ACS) exposure on the rate of hyperbilirubinemia in term newborns.

**Methods:**

This retrospective study was conducted at the Health Sciences University Kayseri Education and Research Hospital, Turkey from June 2017 to June 2018. A total of 6254 pregnant participants aged between 18 and 35 years with a singleton pregnancy were included in the study. The study group included 354 women with low-risk pregnancies (no perinatal risk except threatened preterm labor) who received ACS treatment and were hospitalized because of the threat of preterm labor before the 34^th^ gestational week but delivered after 37 weeks of gestation. The control group was composed of 5900 women with low-risk pregnancies who did not receive ACS treatment throughout their pregnancy and delivered after 37 weeks of gestation.

**Results:**

Maternal age, mean body mass index, gestational week at delivery, nulliparity, previous cesarean history, sex of the baby, fetal weight, labor induction, vaginal delivery, and five minutes. Apgar score were similar in both groups. The neonatal hyperbilirubinemia rate was 20/354 (5.6%) in the ACS treatment group and 564/5900 (9.6%) in the control group.

**Conclusions:**

The neonatal hyperbilirubinemia was significantly decreased in term-born babies exposed to ACS before 34 weeks.

## INTRODUCTION

Neonatal hyperbilirubinemia [physiologic (non-pathologic) jaundice] is a short-lasting benign condition that affects approximately 60% of infants born at term and 80% of preterm infants.^1^ In the first week after birth, increased production of bilirubin combined with decreased bilirubin elimination leads to elevated serum bilirubin concentrations in neonates.^2^,^3^ After the first week, the liver starts to efficiently conjugate and eliminate bilirubin, returning the total serum bilirubin concentration to a normal level. However, some newborns develop severe neonatal hyperbilirubinemia (defined as a plasma bilirubin >25 mg/dL), necessitating hospitalization for phototherapy and exchange transfusion. Kernicterus may result in cerebral palsy or death.^4^

Administering antenatal corticosteroids (ACS) to mothers in cases of impending preterm delivery before 34 weeks of gestation is standard practice.^5^ ACS treatment has been confirmed by systematic reviews of clinical data as being effective at improving preterm delivery outcomes in pregnant women. Synthetic glucocorticoids can promote the rapid maturing of underdeveloped organs in fetuses, and have been demonstrated to be applicable to preventing or reducing the aforementioned complications.^6^ In utero exposure to synthetic glucocorticoids during the critical stages of development can result in altered functioning of various organ systems, which may persist throughout life. For the mother, there is no increased risk of death or chorioamnionitis associated with corticosteroid use.^7^ On the other hand, it has been reported in various studies that ACSs are associated with neonatal hypoglycemia and hyperbilirubinemia.^8^ Today, the effects of ACS administration on perinatal outcomes in the acute period are clearly known but the effects on fetal organ development and neonatal effects in the long-term are still unclear. The present study aimed to evaluate the effects of ACS exposure on the rate of hyperbilirubinemia in term newborns.

## METHODS

The present study was conducted retrospectively at the Health Sciences University Kayseri Education and Research Hospital, was approved by the Ethics Committee of Erciyes University (2018/341), and was carried out in accordance with the Declaration of Helsinki. All data were obtained from hospital data bank and patient files between June 2017 and June 2018.

For this study, two groups were defined. The study group included women with low-risk pregnancy (no perinatal risk except threatened preterm labor) who received ACS treatment and were hospitalized because of the threat of preterm labor before the 34^th^ gestational week, but delivered after 37 weeks of gestation. To decrease the morbidity of preterm delivery, ACS is administered to all pregnant women at 23 to 34 gestational weeks with increased risk for preterm delivery within the next one to seven days as routine clinical practice in our hospital.^5^ A total of 354 pregnant participants aged between 18 and 35 years in a low-risk group with a singleton pregnancy were included in the study group throughout a year.

The control group was composed of women with low-risk pregnancy (no perinatal risk) who did not receive ACS treatment throughout their pregnancy and delivered after 37 weeks of gestation since they do not undergo stress of threated of premature birth during pregnancy. A total of 5900 pregnant participants aged between 18 and 35 years in a low-risk group with a singleton pregnancy between June 2017 and June 2018 were included in the control group of this study.

Exclusion criteria were patients who delivered before 37 gestational weeks or with multiple pregnancies, early membrane rupture, chromosomal or fetal anomaly, Type 1 and 2 diabetes mellitus or gestational diabetes mellitus, chronic hypertension, preeclampsia, collagen vascular disease, and the presence of chronic systemic diseases. Additionally, patients with conditions that may cause neonatal hyperbilirubinemia, such as ABO or Rh(D) incompatibility, hereditary spherocytosis and elliptocytosis, glucose-6-phosphate dehydrogenase deficiency, pruvate kinase deficiency, and congenital erythropoietic porphyria, neonatal polycythemia, inadequate breastfeeding, neonatal sepsis, birth trauma, malnutrition, or other neonatal metabolic diseases were excluded from the study.

In the study group, all pregnant women at 23 to 34 weeks of gestation were hospitalized because of the risk of threatened preterm labor. According to protocol, two doses of 12 mg of betamethasone were administered intramuscularly 24 hours apart.^5^ After the treatment, patients at no risk of acute preterm labor were discharged and routine antenatal follow-up was performed.

Threatened preterm delivery is diagnosed using the clinical criteria of regular painful uterine contractions with cervical dilation and/or effacement. Specifically, at least four uterine contractions per 20 minutes or more than eight in 60 minutes) **combined with** cervical dilation ≥3 cm **or** cervical length <20 mm as measured by transvaginal ultrasound **or** cervical length 20–30 mm determined by transvaginal ultrasound.^9^

According to protocol for surveying newborns for hyperbilirubinemia if risk factors are present, the infant is performed pre-discharge bilirubin testing (total plasma bilirubin level).^10^ The need for bilirubin measurement for routine post-discharge follow-up on postpartum third day is based on the infant’s appearance, weight change and intake, presence of hyperbilirubinemia risk factors, and the pre-discharge total plasma bilirubin (TB) level. In our hospital, the examination of the newborn and measurement of the TB level are routinely performed by a pediatrician in the neonatology policlinic. Neonatal hyperbilirubinemia in infants born at a gestational age of >35 weeks is diagnosed when the total serum or plasma bilirubin levels are higher than the 95^th^ percentile using the hour-specific Bhutani nomogram.^10^ These newborns need hospital care and treatments like phototherapy. TB levels were measured with commercial Microparticle Enzyme Immunoassay (MEIA) kits (Beckman Coulter AU 640 Chemistry Analyzer, Inc. 250 S. Kraemer Blvd. Brea, CA 92821, USA).

The primary outcome of the study was the presence of neonatal hyperbilirubinemia in need of treatment between patients who received ACS owing to threatened preterm labor and who did not. In addition, maternal characteristics, gestational week at delivery, fetal weight, labor induction, vaginal delivery rates, Apgar scores, and length of stay in the neonatal unit were evaluated. The indications for labor induction of both groups were arrest of labor, membrane rupture, oligohydramnios, and necessity of the oxytocin challenge test.

The Shapiro–Wilk test was used to test the normality of the data and the Levene test was used to test the variance homogeneity. Values are expressed as mean ± standard deviation or median (25–75%). Parametric comparisons were made using the t-test, and nonparametric comparisons were made using the Mann–Whitney U test. Categorical data were presented as count and percentage and groups compared using Pearson chi-square tests or Fisher exact tests. Minitab 16 (Minitab Inc.; State College, PA, USA) was used for all analyses. The difference between groups was considered statistically significant when the p value was <0.05.

## RESULTS

Of the 6254 pregnant participants enrolled in the study, their maternal characteristics and fetal outcomes were compared and the results are shown in Tables-I and II. The study group included 354 pregnant participants. In 205 of them TB levels were measured after routine neonatal evaluation, and hyperbilirubinemia was found in 20 (5.6%) of these babies, and were hospitalized for treatment such as phototherapy. The control group included 5900 pregnant participants. In 3245 of them, TB levels were measured after routine neonatal evaluation, and hyperbilirubinemia was found in 564 (9.6%) of these babies, who were hospitalized for treatment such as phototherapy. The neonatal hyperbilirubinemia rate was significantly decreased in the ACS treatment group (p = 0.014). However, length of stay in the neonatal unit was similar in both groups (p = 0.418). Maternal age was 29.0 ± 3.0 years in the ACS treatment group and 28.8 ± 3.1 years in the control group (p = 0.664).

**Table I:** Comparison of maternal characteristics between groups.

	ACS treatment group (n=354)	Control group (n= 5900)	p-value
Maternal age, years	29.0 ± 3.0	28.8 ± 3.1	0.664^&^
Nulliparity, n (%)	121 (34.1%)	2141 (36.3%)	0.521*
BMI, kg/m^2^	27.12 ± 2.08	27.24 ± 2.08	0.727^&^
Previous cesarean history, n (%)	63 (17.7%)	1121 (19%)	0.601*

* Pearson chi-square tests.

& Parametric comparisons were made using the t-test, and nonparametric comparisons were made using the Mann–Whitney U test. Values are expressed as mean ± standard deviation or n (%). BMI, body mass index.

**Table II:** Comparison of delivery characteristics and fetal outcomes between groups.

	ACS treatment group (n= 354)	Control group (n= 5900)	p value
Gestational week at delivery	38.6 ± 0.9	38.7 ± 1.0	0.290^&^
Male sex, n (%)	176 (49.7%)	2714 (46.0%)	0.174*
Fetal weight, g	3190 ± 340	3170 ± 324	0.589^&^
Labor induction, n (%)	105 (29.6%)	1657 (28.1%)	0.522*
Vaginal delivery, n (%)	268 (75.7%)	4602 (78%)	0.316*
5 min Apgar <7, n (%)	1 (0.28%)	34 (0.58%)	0.721*
Neonatal hyperbilirubinemia, n (%)	20 (5.6%)	564 (9.6%)	0.014*
Length of stay in the neonatal unit, day	2.00 ± 0.81	1.69 ± 1.18	0.418^&^

* Pearson chi-square tests.

& Parametric comparisons were made using the t-test, and nonparametric comparisons were made using the Mann–Whitney U test. Values are expressed as mean ± standard deviation or n(%).

## DISCUSSION

The present study aimed to evaluate the effects of ACS treatment applied before 34 weeks of gestation on the rate of hyperbilirubinemia in term newborns and it was found that neonatal hyperbilirubinemia was significantly decreased in term newborns exposed to ACS before 34 weeks of gestation. Both groups in this study were homogenous with respect to maternal characteristics, such as maternal age, nulliparity, BMI, and previous cesarean delivery rate. There were also no significant differences in terms of fetal outcome and delivery characteristics, such as gestational week at delivery, male sex, fetal weight, labor induction, vaginal delivery rate, and 5-minute Apgar score.

To date, few studies have explored the link between gestational ACS treatment and neonatal hyperbilirubinemia. Many authors have reported that betamethasone-exposed infants did not exhibit increased rates of hyperbilirubinemia.^5^ In another study, Nemeth et al. showed that antenatal dexamethasone exposure led to further increased serum unconjugated bilirubin levels in the first week after birth versus non-exposed controls; they also reported a higher rate of hyperbilirubinemia requiring treatment.^11^ Pettit et al. carried out a retrospective cohort study of 6675 preterm deliveries (from 32 to 37 weeks of gestation) and found an association between antenatal betamethasone exposure a neonatal hypoglycemia and hyperbilirubinemia.^8^ Since premature babies have a higher risk of hyperbilirubinemia, the infants delivered before 37 weeks were excluded from the present study.

In this study, the neonatal hyperbilirubinemia was found to be significantly decreased in term-born babies exposed to ACS before 34 gestational weeks. It was also found that ACS exposure reduces hospital care and treatment for the neonatal hyperbilirubinemia (5.6% vs 9.6%). The results could be explained in the context of fetal organ programming.^12^,^13^ Glucocorticoid levels in maternal circulation increase significantly beginning from the twelfth gestational week due to the increased release of corticotropin-releasing hormone (CRH) from maternal hypothalamus. In the third trimester, the levels of glucocorticoids in maternal circulation continue to increase in response to CRH secretion by the placenta. Cortisol is known to suppress secretion of CRH from the hypothalamus, whilst increasing secretion of CRH from the placenta, creating a positive feedback loop that persists until birth.^14^,^15^ The increased glucocorticoid concentration ensures the proper maturation of organs prior to birth in term-born infants and is also the basis for antenatal glucocorticoids therapy. Infants born early will not have been sufficiently exposed to glucocorticoids in utero, and thus the organs will not be sufficiently mature. It is well known that the administration of ACS to women at risk of preterm delivery contributes to the improvement of the heart, liver, fetal brain, kidneys, and gastrointestinal tract by stimulating enzyme activity, protein synthesis, and cell division.^16^

In term newborns, bilirubin production is two to three times higher than adults because neonates have more erythrocytes and fetal erythrocytes have a shorter lifetime (approximately 85 days) than adult erythrocytes. The higher turnover of erythrocytes results in more bilirubin production in newborns. Uridine diphosphogluconurate glucuronosyltransferase (UGT) deficiency means that bilirubin clearance is decreased in newborns: UGT liver activity in term infants at seven days old is approximately one percent of adults, and adult UGT levels are only reached at 14 weeks of age. Moreover, the infant bilirubin load is further increased by increased enterohepatic circulation of bilirubin.^16^ Although the UGT enzyme levels were not measured in the newborns in this study, the positive effects of glucocorticoids on liver development would result in increased UGT enzyme activation.^17^ Increased UGT activation is likely to result in increased bilirubin metabolism and a decrease in neonatal hyperbilirubinemia.

Generally, physiological hyperbilirubinemia resolves spontaneously so that treatment is not required. Bilirubin is neurotoxic at high levels as it is able to cross the blood–brain barrier, resulting in neuronal injury and having adverse effects on long-term neurodevelopment. Thus, a moderate elevation in serum bilirubin levels is often treated to reduce the risk of neurological complications.

### Limitations of this study

This is a retrospective study. The mode of delivery may effect on the neonatal hyperbilirubinemia. However, both groups of this study were homogenous in terms of mode of delivery. In addition, measurement of blood glucose of both maternal and newborn could not be evaluated for this study due to the insufficient hospital data.

The data from the current study show that severe neonatal hyperbilirubinemia was significantly decreased in term-born babies exposed to ACS before 34 weeks. Hence it seems to reduce the need for treatment like phototherapy. Large-scale studies are required to understand the clinical importance of this finding in terms of the long-term consequences and underlying mechanisms of neonatal hyperbilirubinemia.

### Authors’ Contribution:

**ICM** conceived, designed and did statistical analysis & editing of manuscript, is responsible for integrity of research.

**ICM & MES** did data collection and manuscript writing.

**ICM & MES** did review and final approval of manuscript.
